# Effect of Health Expenses on Household Capabilities and Resource Allocation in a Rural Commune in Vietnam

**DOI:** 10.1371/journal.pone.0047423

**Published:** 2012-10-15

**Authors:** Kim Thuy Nguyen, Oanh Thi Hai Khuat, Shuangge Ma, Duc Cuong Pham, Giang Thi Hong Khuat, Jennifer Prah Ruger

**Affiliations:** 1 Yale University School of Medicine, New Haven, Connecticut, United States of America; 2 Institute for Social Development Studies, Hanoi, Vietnam; 3 Biostatistics, Yale University School of Public Health, New Haven, Connecticut, United States of America; Universitat Rovira i Virgili, Spain

## Abstract

**Background:**

Significant health expenses can force households to reduce consumption of items required for daily living and long-term well-being, depriving them of the capability to lead economically stable and healthy lives. Previous studies of out-of-pocket (OOP) and other health expenses have typically characterized them as “catastrophic” in terms of a threshold level or percentage of household income. We aim to re-conceptualize the impact of health expenses on household “flourishing” in terms of “basic capabilities.”

**Methods and Findings:**

We conducted a 2008 survey covering 697 households, on consumption patterns and health treatments for the previous 12 months. We compare consumption patterns between households with and without inpatient treatment, and between households with different levels of outpatient treatment, for the entire study sample as well as among different income quartiles. We find that compared to households without inpatient treatment and with lower levels of outpatient treatment, households with inpatient treatment and higher levels of outpatient treatment reduced investments in basic capabilities, as evidenced by decreased consumption of food, education and production means. The lowest income quartile showed the most significant decrease. No quartile with inpatient or high-level outpatient treatment was immune to reductions.

**Conclusions:**

The effects of health expenses on consumption patterns might well create or exacerbate poverty and poor health, particularly for low income households. We define health expenditures as catastrophic by their reductions of basic capabilities. Health policy should reform the OOP system that causes this economic and social burden.

## Introduction

With out-of-pocket payments (OOP) accounting for 68% of total health expenditures in 2005, Vietnam’s healthcare system presents substantial financial hardships for its primarily low income population [1]. Vietnam had established a state-run health system that provided free universal access, after the reunification of the country under Socialist Party rule in 1975. Financing for this system had been indirectly supported by economic aid from the Soviet Union, which was terminated by the dissolution of the Soviet Union in 1990 [2]. In the late 1980s, Vietnam began Doi Moi (“New Era”), economic reforms which, among its other effects, legalized market enterprises in healthcare. Severe underfunding of the public healthcare system was accompanied by the development of a user-fee system in public health facilities and increased demand for unofficial payments by public health workers. By 2002, 72% of total health expenditures went to the private sector [2], [3]. A study of catastrophic (defined as OOP exceeding 40% of a household’s income after meeting subsistence costs) health expenditures in 89 countries ranked Vietnam at the top, with the highest proportion of households with catastrophic payments [4].

The WHO acknowledges that defining health costs as “catastrophic” based solely on the percentage of income they constitute does not determine the long-term impact of health events [5]. Our study is grounded in an alternative framework of financial protection, a multidimensional approach that quantitatively assesses important dimensions and their interrelations from the households’ perspective [6].

In Vietnam, where low-income households spend 22% of their income on healthcare, a household’s financial choices might depend on health expenditures [7]. Studies in other countries suggest that when low-income rural patients pay for healthcare primarily out-of-pocket, income and savings are insufficient to cover health costs [8]. Instead, patients reduce consumption of food, education, farming expenses and other production means [7], [9], [10].

According to the 1998 Vietnam Living Standard Survey the price of one admission to a public hospital equaled 60% of annual non-food expenditure for low- or middle-income patients [11]. Following such a health shock, Vietnamese households spend less on food [12]. Less costly health services can also pose difficulties for poor households. One study found that treatment for multiple minor illnesses accounted for the majority of healthcare spending [13], while another study revealed that the proportion of a household’s total health expenditure on medications was nearly five times that on inpatient treatment [14].

A catastrophic threshold is difficult to define. The WHO has recognized that there is no general consensus for a threshold and that any choice of one requires assessment of relativity in terms of contextual factors [4]. A multi-dimensional framework is required [6]. We assert that in a vulnerable population like Dai Dong, where over half of the households live at or below the international poverty standard of $1.25/day per capita [15], even small reductions in any dimension of functionings can be meaningful and problematic because they reduce individuals’ ability to be well-nourished, to gain employment, and to be educated. Thus, in this study, we’ve focused on identifying which consumptions decrease the most, quantifying this decrease, and determining the manner and degree to which health expenses may affect a household’s overall flourishing.

## Methods

### Ethics Approval

This study was approved by the Human Investigation Committee at Yale University and the Institutional Review Board at the Institute of Social Development Studies in Hanoi, Vietnam. Written consent was attained from every participant. We had an HIC exemption for the project.

### Data Collection

As three quarters of Vietnam’s population and 90% of its poor residents live in rural areas, we conducted our survey in Dai Dong, a rural commune 35 km west of Hanoi. An average rural commune contains 2000–2500 households, 15% of whom are poor or near-poor, with 8000–10000 people [16]. With 2230 households, 15.7% of whom are poor or near-poor, and a population of 9678, Dai Dong represents an average rural commune. Dai Dong is broadly comparable to rural Vietnam more generally in other respects, such as gender composition (51.5% and 49.5% male, respectively) and household size (3.8 and 4.3 members). The mean annual income per capita in Dai Dong (US$631) lies between that of the Red River Delta region (US$744) – to which Dai Dong belongs – and rural Vietnam (US$541), which covers the poorest areas of the country.

Households were classified as poor, near-poor, or “other” by the Dai Dong commune administration based on cut-offs for per capita annual income, in accordance with the policies and standards set by the national Ministry of Labour, Invalids and Social Affairs [17]. We selected all households designated as poor (166) and near-poor (184). We randomly selected an equal number (356) of households designated as “other”, distributed equally among Dai Dong’s 11 villages and representing 18.9% of “other” households. Such a sampling strategy allowed us to investigate inequities among different income levels, and guaranteed that poor and near-poor households are sufficiently represented.

In July 2008 we surveyed 706 households comprising 2697 people, with a response rate of near 100%. The survey comprised four parts, spanning a time period of 12 months prior to the survey date. Part 1 requested demographic information for household members. Part 2 collected data on household income from itemized sources: wages/salaries, crops, livestock, trade (businesses and markets), gifts from relatives (a cultural practice), interest on savings, and other sources (such as gambling and loan interest). Part 3 included itemized household expenditures: food, education, production means (items for farming, trade, or other business), transportation, construction, charity (gifts for mourning, when community members experience loss), durable goods, utilities, daily goods, social activities, insurance (e.g., health and property), gifts (assistance to relatives and friends), tobacco/alcohol, loan interest (paid on loans in the past 12 months, regardless of when the loan was borrowed), healthcare, and other items. Data on healthcare expenditure were collected for each episode of inpatient treatment and all episodes of outpatient treatments for each household member in the previous 12 months. Because inpatient treatments were relatively infrequent, recall for expenditures for each episode is likely to be more accurate. We asked respondents for expenditures for all outpatient treatments because there were often multiple outpatient treatments, and we found in the pilot study that households had an easier time remembering how they paid for all episodes generally rather than how they paid for each separate episode. Health expenditure for inpatient and outpatient treatment included direct medical costs (payments made to the health facility for services and treatment); direct other medical costs (payments for additional medicines and supplies outside the health facility); unofficial fees for health workers; gifts for those who assisted in procuring and administering care; food for the patient and escorts during the medical visit; and income lost by the patient and caretakers in the household to illness and treatment. Full details of health expenditures are reported elsewhere [18].

Part 4 sought information regarding total number of illness episodes, and inpatient and outpatient treatments. A household member was considered ill if he or she was: diagnosed as ill by a healthcare professional, experienced discomfort, or unable to pursue usual activities. Inpatient treatment was defined as an appointment, procedure and/or treatment requiring an overnight stay in a health facility. Outpatient treatment was defined similarly but without an overnight stay. Outpatient treatment included services and medicine administered by a hospital, commune health clinic, private health facility, or village health worker. Households also noted if they did not treat an episode of illness.

### Analysis

Analysis was conducted using S-Plus. Nine records out of 706 had inconsistent measurements; these we excluded from analysis to yield an effective sample size of 697. Summary statistics were obtained for the whole cohort and for income quartiles with equal sample sizes based on total self-reported income for subset analyses (Table 1).

**Table 1:** Household characteristics by income quartile.

Variables	Descriptions	Total	Income Quartile						
			1	2	3		4		
***Data on household head***									
Age	Mean age	49.6	53.3	48.4		48.5		48.2	
Gender	Percentage female	49.5	55.9	50.0		46.5		45.4	
Marital status	Percentage married	76.9	67.8	73.0		82.6		84.5	
	Percentage unmarried	4.2	6.2	5.2		2.3		2.9	
	Percentage divorced/widowed	18.7	26	21.8		15.1		11.5	
Education status	Mean number of years	7.7	6.5	7.5		8.3		8.8	
Occupation	Percentage of farmers	49.8	49.7	53.4		48.8		47.1	
***Data on household***									
Household size	Mean number of household members	3.8	3.8	4.1		3.9		3.6	
Older than 65	Percentage with ≥1 member of age >65 years	27.0	37.9	25.3		25.0		19.5	
Younger than 18	Percentage with ≥1 member of age <18 years	61.8	61.0	67.8		65.7		52.9	
Inpatient	Percentage with ≥1 inpatient treatment	22.5	22.0	25.3		22.7		20.1	
Outpatient Level 1	Percentage with 0–4 outpatient treatments	58.5	56.5	56.9		58.1		62.6	
Outpatient Level 2	Percentage with 5–10 outpatient treatments	25.0	26.0	25.9		26.7		21.3	
Outpatient Level 3	Percentage with >10 outpatient treatments	16.5	17.5	17.2		15.1		16.1	
Sample size	Total number of households	697	177	174		172		174	

*Note:* Households were stratified into income quartiles, with Quartile 1 as the lowest and Quartile 4 as the highest income quartile.

We created two variables to represent inpatient and outpatient treatment status. For inpatient, we created a binary variable indicating zero versus at least one inpatient treatment (few households had multiple inpatient treatments). For outpatient, to reflect the possible effect of accumulation, we created a categorical variable with three levels representing treatment intensity. Level 1∶0–4 treatments; Level 2∶5–10; Level 3: >10.

We conducted multiple regression analysis, looking at how the percentage of each type of consumption varied by healthcare expenditure. We adjusted for age, gender, marital status, occupation and education of household head, household size, and presence of young and elderly household members. We denote 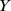
as the percentage of one type of consumption, and 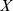
as the covariates. The model assumes that 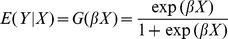.

We also conducted multiple regression analyses of opportunity cost, adjusting for the same variables above. We estimated the impact of a 1% increase in medical expenses in terms of a change in the percentage of other allocation categories. Then, with a 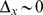 change in *X*, the expected change in *Y* is 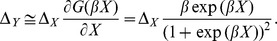 This calculation is valid as long as 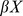
is bounded and 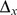
is sufficiently small. For all regression models, we conducted model diagnosis and found no obvious lack of fit.

## Results

We present household characteristics by income quartile in Table 1. Comparing quartiles 1 and 4, quartile 1 had fewer married household heads (67.8% vs 84.5%), more female household heads (55.9%, vs 45.4%), fewer years of education (6.5 years vs 8.8 years), and more dependents (37.9% vs 19.5% had at least one member older than 65, and 61.0% vs 52.9% had at least one member younger than 18). Among all quartiles, the proportions of households with inpatient treatment and Level 3 outpatient treatment are similar. Unlike previous household surveys where the percentage of the sample population having an illness episode ranges from 35.1% [10] to 62.4% [14], 83.6% of our sampled households had at least one illness that warranted treatment. Among 697 households, 612 (87.8%) had no untreated episodes of illness and 664 (97.5%) had five or fewer untreated episodes of illness.

Table 2 shows the mean amount and percentage of household budget spent on allocation categories among income quartiles. Tables 3, 4, 5 reveal the correlation between healthcare expenses and other consumptions. Table 3 shows the unadjusted mean and percentage differences in expenditures between households with and without inpatient treatment, and between households with Level 1 and higher levels of outpatient treatment. Our multiple regression analysis (Table 4) adjusts for household characteristics.

**Table 2:** Mean household allocations by income quartiles.

		Total		Income Quartile 1		Income Quartile 2		Income Quartile 3		Income Quartile 4	
Allocation category	Description	Mean	Percent	Mean	Percent	Mean	Percent	Mean	Percent	Mean	Percent
Food	Rice, produce, meat, etc.	3614.1	28.9	2940.7	41.7	3323.2	37.7	3798.3	34.0	4407.7	19.1
Education	Tuition, books, room and board	904.2	7.2	519.1	7.4	847.7	9.6	1103.5	9.9	1155.3	5.0
Productionmeans	Items for farming, business,trade	2647.7	21.2	519.5	7.4	670.4	7.6	1498.3	13.4	7913.8	34.2
Transportation	Gas, oil, repairs, motorcycles	299.4	2.4	136.3	1.9	240.2	2.7	317.8	2.8	506.3	2.2
Healthcare	Treatment, medicine, gifts to health staff	732.8	5.9	736.6	10.4	623.3	7.1	826.6	7.4	746.4	3.2
Construction	Building and repair of home or business	1755.9	14	956.5	13.6	1353.4	15.4	1164.5	10.4	3556	15.4
Charity	Gifts for mourning, forcommunity	514.7	4.1	327.7	4.6	380.0	4.3	391.6	3.5	961.5	4.2
Durable goods	Furniture, electronic devices	472.7	3.8	114.7	1.6	282.4	3.2	450.1	4.0	1049.4	4.5
Utilities	Electricity, gas, water, phone	470.1	3.8	286.8	4.1	377.5	4.3	480.5	4.3	738.9	3.2
Daily goods	Toiletries, kitchen supplies	237.3	1.9	202.3	2.9	230.4	2.6	262.0	2.3	255.3	1.1
Social activities	Entertainment, travel, weddings, holidays	30.6	0.2	12.4	0.2	26.8	0.3	17.9	0.2	65.6	0.3
Insurance	Property, health, etc.	20.3	0.2	9.3	0.1	20.1	0.2	20.6	0.2	31.6	0.1
Gifts	Offerings to family and friends	21.3	0.2	20.2	0.3	13.7	0.2	17.9	0.2	33.4	0.1
Tobacco/Alcohol	Cigarettes, tobacco, liquor	137.7	1.1	85.4	1.2	109.9	1.2	160.6	1.4	195.9	0.8
Loan interest	Interest paid on loans	131.9	1.1	89.5	1.3	53.6	0.6	187.7	1.7	198	0.9
Other	Expenditures not listed above	572.9	4.6	93.0	1.3	257.3	2.9	492.7	4.4	1456.2	6.3
Total		12517.5	100.0	7055.3	100.0	8806.2	100.0	11180.9	100.0	23106.2	100.0

*Note:* Data are reported in 1000 VND.

**Table 3:** Unadjusted differences in allocation categories between households with and without inpatient treatment, and between households with and without outpatient treatment.

INPATIENT TREATMENT											
Difference between households with and without inpatient treatment											
		Total		Income Quartile 1		Income Quartile 2		Income Quartile 3		Income Quartile 4	
Allocation category		Mean	Percentage	Mean	Percentage	Mean	Percentage	Mean	Percentage	Mean	Percentage
Food		−233.7	−2.0	−299.2	−15.8	−530.8	−1.3	−17.7	0.4	98.2	−0.6
Education		−27.7	−0.3	−24.3	−2.4	−203.3	−1.2	101.2	1.1	69.2	0.0
Production means		−67.2	−0.6	−193.2	−4.5	−89.0	0.0	−336.7	−2.8	1476.9	4.5
Transportation		34.6	0.3	12.9	−0.4	3.3	0.4	−11.1	−0.1	180.4	0.6
Healthcare		1008.6	8.1	617.3	4.4	842.2	11.2	1150.9	10.5	1510.7	6.2
Construction		−112.6	−0.9	2076.3	21.2	−870.4	−8.5	−588	−5.2	−770.6	−4.0
Charity		−90.4	−0.8	67.7	−0.5	−126.4	−0.9	5.4	0.1	−238.3	−1.2
Durable goods		−210.3	−1.7	110.1	0.8	−154.5	−1.4	−280.9	−2.5	−441.8	−2.1
Utilities		30.9	0.2	−16.6	−1.4	77.3	1.6	51.2	0.5	57.2	0.1
Daily goods		23.0	0.1	2.9	−0.8	64.5	1.1	56.3	0.6	−36.7	−0.2
Social activities		9.8	0.1	18.8	0.2	−30.7	−0.4	16.7	0.2	46.0	0.2
Insurance		2.3	0.0	6.5	0.1	5.3	0.1	−6.6	−0.1	5.2	0.0
Gifts		−3.7	−0.1	6.2	0.0	−4.6	−0.1	0.7	0.0	−15.0	−0.1
Tobacco/Alcohol		44.0	0.4	32.2	0.0	−61.3	−0.6	168.5	1.5	58.4	0.2
Loan interest		25.4	0.2	−11.4	−0.5	−13.5	−0.1	−68.2	−0.6	233.6	0.9
Other		−193.5	−1.5	15.1	−0.2	−27.4	0.1	−374.6	−3.3	−249.6	−1.4
**OUTPATIENT TREATMENT**											
**Difference between households with and without outpatient treatment**											
		**Total**		**Income Quartile 1**		**Income Quartile 2**		**Income Quartile 3**		**Income Quartile 4**	
**Allocation category**	**Level**	**Mean**	**Percentage**	**Mean**	**Percentage**	**Mean**	**Percentage**	**Mean**	**Percentage**	**Mean**	**Percentage**
Food	2	−372.2	2.0	54.7	−12.0	−347.3	2.0	43.8	2.0	−1133.3	1.1
	3	−347.5	−2.0	−120.4	−16.0	−416.8	3.0	−560.5	4.0	−127.6	−1.6
Education	2	−284.0	−1.0	40.0	−1.0	−312.6	−2.0	−601.9	−5.0	−187.4	0.9
	3	−270.2	−2.0	36.8	−2.0	−246.8	0.0	−702.4	−5.0	−131.7	−0.8
Production means	2	−567.8	−1.0	470.0	4.0	47.9	2.0	−528.0	−4.0	−890.5	7.6
	3	257.0	3.0	−55.2	−3.0	431.6	7.0	−928.0	−6.0	2345.4	7.3
Transportation	2	−30.7	0.0	74.8	1.0	8.6	0.0	−47.9	0.0	−109.8	0.3
	3	43.9	1.0	216.3	2.0	−1.5	0.0	−51.3	0.0	41.5	0.1
Healthcare	2	50.4	2.0	100.3	−1.0	−296.3	−3.0	154.4	2.0	299.5	2.9
	3	218.5	2.0	865.1	8.0	−159.0	−1.0	338.6	6.0	−188.7	−0.9
Construction	2	−708.7	−3.0	1176.7	13.0	−250.5	0.0	215.5	2.0	−3875.5	−14.9
	3	−174.8	−1.0	1536.4	16.0	−1296.0	−12.0	−164.1	1.0	−516.9	−3.0
Charity	2	−252.5	−2.0	1.5	−2.0	−139.5	−1.0	1.9	1.0	−803.2	−2.5
	3	−281.0	−2.0	−118.6	−3.0	−192.0	−2.0	−13.1	1.0	−721.7	−3.1
Durable goods	2	−8.2.0	0.0	89.5	1.0	58.0	1.0	−150.4	−1.0	148.5	2.5
	3	−56.2	−1.0	95.3	1.0	50.7	1.0	−387.7	−3.0	83.0	0.1
Utilities	2	−67.5	0.0	28.5	−1.0	−35.1	0.0	−14.2	0.0	−198.8	0.1
	3	79.5	0.0	4.5	−2.0	114.9	2.0	10.9	1.0	242.7	0.8
Daily goods	2	−37.4	0.0	−10.8	−1.0	−93.3	−1.0	−15.5	0.0	−22.3	0.3
	3	−34.3	0.0	21.0	0.0	−78.5	−1.0	5.9	1.0	−78.7	−0.3
Social activities	2	−3.7	0.0	−4.6	0.0	−14.7	0.0	25.9	0.0	−16.0	0.0
	3	17.0	0.0	8.0	0.0	−3.1	0.0	−7.6	0.0	74.0	0.3
Insurance	2	−6.1	0.0	0.6	0.0	−13.1	0.0	−2.1	0.0	−6.8	0.0
	3	2.1	0.0	7.2	0.0	−14.9	0.0	8.7	0.0	10.2	0.1
Gifts	2	−0.4	0.0	12.5	0.0	−4.5	0.0	3.3	0.1	−10.8	0.0
	3	−3.5	0.0	−5.2	0.0	−3.3	0.0	6.5	0.1	−9.1	−0.1
Tobacco/	2	−4.2	0.0	−24.4	0.0	55.6	1.0	15.6	0.2	−47.5	0.1
Alcohol	3	28.7	0.0	37.9	0.0	116.6	2.0	50.0	0.9	−76.9	−0.4
Loan interest	2	−5.3	0.0	85.9	1.0	−26.0	−1.0	−90.0	−0.7	25.4	0.4
	3	−2.0	0.0	−14.4	0.0	−22.4	0.0	−67.1	−0.2	116.6	0.4
Other	2	−106.0	0.0	−10.7	−1.0	−116.6	0.0	260.1	2.6	−357.2	0.4
	3	−125.0	−1.0	−50.9	−1.0	−196.4	−1.0	−294.6	−2.1	175.1	0.3

*Note*: Data for mean differences are reported in 1000 VND.

**Table 4:** Results of multivariate regression analyses of inpatient and outpatient treatment and percentage of household allocation categories.

INPATIENT TREATMENT									
Allocation categories		Total		Income Quartile 1		Income Quartile 2		Income Quartile 3	Income Quartile 4
Food		−0.097 (0.002)****		−0.575 (0.004)****		−0.001 (0.004)		0.059 (0.004)****	0.097 (0.004)****
Education		−0.164 (0.003) ****		−0.503 (0.009)****		−0.196 (0.007)****		0.122 (0.006)****	0.069 (0.007)****
Production means		−0.152 (0.002) ****		−0.838 (0.010)****		−0.151 (0.008)****		−0.037 (0.006)****	−0.125 (0.003)****
Transportation		0.080 (0.005) ****		−0.402 (0.016)****		0.046 (0.012)****		0.100 (0.011)****	0.338 (0.009)****
Healthcare		1.324 (0.003) ****		0.784 (0.007)****		1.385 (0.007)****		1.305 (0.006)****	1.891 (0.007)****
Construction		−0.105 (0.003) ****		1.244 (0.006)****		−0.917 (0.007)****		−0.977 (0.008)****	−0.600 (0.005)****
Charity		−0.030 (0.004) ****		−0.032 (0.010)***		−0.075 (0.010)****		0.021 (0.010)**	0.279 (0.008)****
Durable goods		−0.635 (0.005) ****		0.474 (0.015)****		−0.564 (0.013)****		−0.852 (0.012)****	−0.570 (0.008)****
Utilities		0.013 (0.004)***		−0.428 (0.012)****		0.386 (0.009)****		0.146 (0.009)****	0.016 (0.008)**
Daily goods		0.088 (0.006) ****		−0.340 (0.014)****		0.448 (0.012)****		0.280 (0.011)****	−0.290 (0.015)****
Social activities		0.204 (0.016) ****		0.678 (0.048)****		−2.24 (0.082)****		0.238 (0.045)****	0.591 (0.023)****
Insurance		0.014 (0.020)		0.273 (0.054)****		0.293 (0.040)****		−0.252 (0.047)****	0.321 (0.037)****
Gifts		−0.124 (0.021) ****		0.136 (0.04)***		−0.211 (0.054)****		0.066 (0.046)	−0.680 (0.044)****
Tobacco/Alcohol		0.321 (0.008) ****		0.020 (0.019)		−0.759 (0.021)****		1.170 (0.013)****	0.020 (0.016)
Interest on loans		0.071 (0.008) ****		−0.486 (0.021)****		−0.126 (0.027)****		−0.830 (0.016)****	0.564 (0.014)****
Other		−0.201 (0.005) ****		−0.137 (0.019)****		−0.001 (0.012)****		−1.450 (0.012)****	0.290 (0.006)****
**OUTPATIENT TREATMENT**									
**Allocation categories**	**Level**	**Total**	**Income Quartile 1**		**Income Quartile 2**		**Income Quartile 3**		**Income Quartile 4**
Food	2	0.102 (0.002)****	−0.314 (0.004)****		0.236 (0.004)****		−0.045 (0.004)****		−0.070 (0.004)****
	3	−0.061 (0.002)****	−0.467 (0.005)****		0.290 (0.005)****		0.111 (0.005)****		−0.192 (0.004)****
Education	2	−0.256 (0.004)****	−0.280 (0.009)****		−0.330 (0.007)****		−0.415 (0.007)****		−0.516 (0.008)****
	3	−0.566 (0.004)****	−0.595 (0.010)****		−0.462 (0.008)****		−0.397 (0.009)****		−0.767 (0.007)****
Production means	2	0.072 (0.002)****	0.284 (0.008)****		0.318 (0.008)****		−0.118 (0.006)****		0.594 (0.003)****
	3	0.239 (0.002)****	−0.842 (0.012)****		0.809 (0.008)****		−0.403 (0.008)****		0.438 (0.003)****
Transportation	2	−0.022 (0.006)****	0.181 (0.017)****		0.163 (0.012)****		−0.004 (0.011)****		−0.278 (0.010)****
	3	−0.032 (0.006)****	0.553 (0.016)****		0.097 (0.014)****		0.119 (0.014)****		−0.409 (0.010)****
Healthcare	2	0.304 (0.004)****	0.035 (0.008)****		−0.008 (0.009)****		0.172 (0.007)****		0.743 (0.007)****
	3	0.501 (0.004)****	1.004 (0.008)****		0.393 (0.010)****		0.746 (0.008)****		−0.604 (0.010)****
Construction	2	−0.376 (0.003)****	1.762 (0.009)****		−0.506 (0.006)****		0.218 (0.006)****		−2.370 (0.008)****
	3	−0.155 (0.003)****	2.140 (0.010)****		−1.748 (0.011)****		−0.037 (0.008)****		−0.216 (0.004)****
Charity	2	−0.231 (0.005)****	−0.233 (0.010)****		−0.100 (0.011)****		−0.098 (0.010)****		−0.829 (0.009)****
	3	−0.307 (0.006)****	−0.593 (0.014)****		−0.070 (0.013)****		0.242 (0.012)****		−0.426 (0.010)****
Durable goods	2	0.132 (0.004)****	0.256 (0.017)****		0.132 (0.011)****		−0.275 (0.010)****		0.399 (0.007)****
	3	−0.207 (0.005)****	0.177 (0.019)****		0.255 (0.013)****		−1.017 (0.016)****		−0.133 (0.008)****
Utilities	2	−0.004 (0.005)****	−0.264 (0.011)****		0.104 (0.011)****		0.082 (0.009)****		−0.154 (0.009)****
	3	0.104 (0.005)****	−0.472 (0.013)****		0.595 (0.011)****		0.230 (0.011)****		−0.055 (0.008)****
Daily goods	2	−0.032 (0.006)****	−0.326 (0.013)****		−0.252 (0.014)****		−0.086 (0.012)****		0.196 (0.013)****
	3	−0.159 (0.007)****	−0.012 (0.016)****		−0.101 (0.015)****		0.183 (0.014)****		−0.426 (0.016)****
Social activities	2	−0.017 (0.018)****	−0.754 (0.063)****		0.468 (0.048)****		1.021 (0.046)****		−0.669 (0.033)****
	3	0.364 (0.018)****	0.469 (0.059)****		0.863 (0.044)****		−0.513 (0.101)****		−0.318 (0.025)****
Insurance	2	−0.144 (0.023)****	−0.179 (0.063)****		−0.588 (0.049)****		−0.226 (0.044)****		0.093 (0.043)****
	3	−0.008 (0.023)****	0.241 (0.064)****		−0.629 (0.060)****		0.454 (0.046)****		−0.180 (0.039)****
Gifts	2	0.249 (0.020)****	0.575 (0.039)****		0.069 (0.056)****		0.234 (0.045)****		0.152 (0.039)****
	3	0.115 (0.026)****	0.154 (0.064)****		0.242 (0.061)****		0.707 (0.052)****		−0.353 (0.044)****
Tobacco/Alcohol	2	0.078 (0.008)****	−0.684 (0.023)****		0.720 (0.018)****		0.362 (0.015)****		−0.398 (0.016)****
	3	0.168 (0.009)****	−0.439 (0.021)****		0.968 (0.018)****		0.684 (0.017)****		−0.687 (0.019)****
Loan interest	2	0.096 (0.008)****	0.268 (0.019)****		−0.095 (0.030)****		0.011 (0.017)****		−0.147 (0.017)****
	3	−0.053 (0.010)****	−0.744 (0.029)****		−0.428 (0.032)****		0.201 (0.019)****		0.228 (0.015)****
Other	2	0.216 (0.004)****	−0.474 (0.019)****		−0.154 (0.013)****		0.803 (0.009)****		0.281 (0.007)****
	3	0.162 (0.005)****	−1.323 (0.027)****		−0.644 (0.018)****		−0.833 (0.016)****		0.707 (0.006)****

*Note*: Robust standard errors in parentheses. This table depicts estimated regression coefficients from logistic models, after controlling for household size, age, gender, marital status, occupation and education of household head, presence of household member under 18 years or over 65 years old.

* Significant at p<0.10.

** Significant at p<0.05.

*** Significant at p<0.01.

**** Significant at p<0.001.

### Inpatient Treatment

Households with treatment in quartile 1 (poorest) showed the greatest reduction in food (14.0%), production means (5.7%), and education (3.4%) (p<0.001). Quartile 2 decreased most in construction (11.9%), education (1.7%), and durable goods (1.8%) (p<0.001). Quartile 3 and 4 showed the largest reduction in construction (9.1% and 7.8%). (Table 5) In contrast to quartiles 1 and 2, quartile 3 increased food (1.3%) and education (1.1%). Like quartiles 1 and 2, quartile 3 decreased production means, though to a lesser degree (0.4%) (p<0.001). For quartile 4, the greatest decreases occurred in construction (7.8%), production means (2.8%), and durable goods (2.5%) (p<0.001). Similar to quartile 3, quartile 4 households with inpatient treatment increased food (1.5%) and slightly increased education (0.3%) (p<0.001).

**Table 5:** Adjusted mean and percentage differences in allocation categories between households with and without inpatient treatment, and outpatient treatment.

INPATIENT TREATMENT											
Difference between households with and without inpatient treatment											
		Total		Income Quartile 1		Income Quartile 2		Income Quartile 3		Income Quartile 4	
Allocation category		Mean	Percentage	Mean	Percentage	Mean	Percentage	Mean	Percentage	Mean	Percentage
Food		−248.7	−2.0	−986.0	−14.0	−1.7	0.0	148.5	1.3	347.5	1.5
Education		−137.4	−1.1	−242.1	−3.4	−150.1	−1.7	121.3	1.1	75.9	0.3
Production means		−317.0	−2.5	−403.2	−5.7	−93.8	−1.1	−48.6	−0.4	−649.9	−2.8
Transportation		23.4	0.2	−53.7	−0.8	10.8	0.1	31.0	0.3	167.5	0.7
Healthcare		913.8	7.3	517.1	7.3	802.1	9.1	999.2	8.9	1365.8	5.9
Construction		−158.3	−1.3	1028.6	14.6	−1050.8	−11.9	−1018.9	−9.1	−1804.8	−7.8
Charity		−14.7	−0.1	−10.0	−0.1	−27.3	−0.3	8.1	0.1	256.7	1.1
Durable goods		−289.0	−2.3	53.5	0.8	−154.3	−1.8	−367.9	−3.3	−570.9	−2.5
Utilities		6.0	0.0	−117.7	−1.7	139.5	1.6	67.4	0.6	11.1	0.0
Daily goods		20.4	0.2	−66.8	−1.0	100.5	1.1	71.5	0.6	−73.2	−0.3
Social activities		6.2	0.0	8.4	0.1	−59.8	−0.7	4.2	0.0	38.6	0.2
Insurance		0.3	0.0	2.5	0.0	5.9	0.1	−5.2	0.0	10.1	0.0
Gifts		−2.6	0.0	2.7	0.0	−2.9	0.0	1.2	0.0	−22.7	−0.1
Tobacco/Alcohol		43.7	0.3	1.7	0.0	−82.4	−0.9	185.1	1.7	4.0	0.0
Loan interest		9.3	0.1	−42.9	−0.6	−6.7	−0.1	−153.1	−1.4	110.7	0.5
Other		−109.9	−0.9	−12.6	−0.2	−0.2	0.0	−683.0	−6.1	395.5	1.7
**OUTPATIENT TREATMENT**											
**Difference between households with and without outpatient treatment**											
		**Total**		**Income Quartile 1**		**Income Quartile 2**		**Income Quartile 3**		**Income Quartile 4**	
**Allocation category**	**Level**	**Mean**	**Percentage**	**Mean**	**Percentage**	**Mean**	**Percentage**	**Mean**	**Percentage**	**Mean**	**Percentage**
Food	2	262.8	2.1	−539.0	−7.6	487.5	5.5	−112.1	−1.0	−250.6	−1.1
	3	−157.5	−1.3	−800.6	−11.4	600.4	6.8	279.2	2.5	−684.8	−3.0
Education	2	−214.4	−1.7	−134.9	−1.9	−252.5	−2.9	−412.6	−3.7	−566.5	−2.5
	3	−475.1	−3.8	−286.1	−4.1	−353.7	−4.0	−395.0	−3.5	−841.7	−3.6
Production means	2	149.3	1.2	136.6	1.9	197.2	2.2	−152.7	−1.4	3091.0	13.4
	3	498.9	4.0	−405.1	−5.7	501.2	5.7	−522.5	−4.7	2277.1	9.9
Transportation	2	−6.3	−0.1	24.2	0.3	38.1	0.4	−1.1	0.0	−137.7	−0.6
	3	−9.3	−0.1	74.0	1.0	22.7	0.3	36.7	0.3	−202.5	−0.9
Healthcare	2	209.5	1.7	23.0	0.3	−4.4	−0.1	131.3	1.2	536.4	2.3
	3	345.9	2.8	662.6	9.4	227.6	2.6	571.0	5.1	−436.0	−1.9
Construction	2	−568.0	−4.5	1457.1	20.7	−579.8	−6.6	227.2	2.0	−7130.9	−30.9
	3	−233.7	−1.9	1769.5	25.1	−2002.7	−22.7	−38.5	−0.3	−651.0	−2.8
Charity	2	−114.1	−0.9	−72.7	−1.0	−36.3	−0.4	−36.9	−0.3	−764.0	−3.3
	3	−151.3	−1.2	−185.3	−2.6	−25.4	−0.3	91.4	0.8	−392.6	−1.7
Durable goods	2	60.2	0.5	28.8	0.4	36.1	0.4	−118.8	−1.1	400.2	1.7
	3	−94.4	−0.8	20.0	0.3	69.8	0.8	−439.1	−3.9	−132.8	−0.6
Utilities	2	−1.7	0.0	−72.6	−1.0	37.6	0.4	37.6	0.3	−110.5	−0.5
	3	47.1	0.4	−129.8	−1.8	215.2	2.4	105.7	0.9	−39.5	−0.2
Daily goods	2	−7.5	−0.1	−64.1	−0.9	−56.4	−0.6	−22.0	−0.2	49.4	0.2
	3	−37.0	−0.3	−2.4	0.0	−22.8	−0.3	46.7	0.4	−107.6	−0.5
Social activities	2	−0.5	0.0	−9.3	−0.1	12.5	0.1	18.2	0.2	−43.7	−0.2
	3	11.1	0.1	5.8	0.1	23.0	0.3	−9.2	−0.1	−20.8	−0.1
Insurance	2	−2.9	0.0	−1.7	0.0	−11.8	−0.1	−4.6	0.0	2.9	0.0
	3	−0.2	0.0	2.2	0.0	−12.6	−0.1	9.3	0.1	−5.7	0.0
Gifts	2	5.3	0.0	11.6	0.2	0.9	0.0	4.2	0.0	5.1	0.0
Tobacco/	3	2.5	0.0	3.1	0.0	3.3	0.0	12.7	0.1	−11.8	−0.1
Alcohol	2	10.6	0.1	−57.7	−0.8	78.1	0.9	57.3	0.5	−77.4	−0.3
	3	22.9	0.2	−37.0	−0.5	105.1	1.2	108.2	1.0	−133.5	−0.6
Loan interest	2	12.5	0.1	23.7	0.3	−5.1	−0.1	2.1	0.0	−28.8	−0.1
	3	−6.9	−0.1	−65.7	−0.9	−22.8	−0.3	37.1	0.3	44.7	0.2
Other	2	117.9	0.9	−43.5	−0.6	−38.4	−0.4	378.4	3.4	383.9	1.7
	3	88.8	0.7	−121.4	−1.7	−160.9	−1.8	−392.3	−3.5	964.7	4.2

*Note*: This table depicts results after controlling for household size, age, gender, marital status, occupation and education of household head, presence of household member under 18 years or over 65 years old.

### Outpatient Treatment

Table 5 shows the differences in expenditures among households with different levels of outpatient treatment. Compared to households with Level 1 (lowest) outpatient treatment, households with Level 2 had the greatest decrease in budget percentage on construction (4.5%), education (1.7%), and charity (0.9%) (p<0.001). They increased food (2.1%) and production means (1.2%) (p<0.001). Level 3 (highest) decreased most on education (3.8%), construction (1.9%), and food (1.3%) (p<0.001). Production means increased (4.0%).

For income quartile 1, as outpatient treatment levels increased, consumption of food, education, charity, and utilities decreased most. In households with Level 3 outpatient treatments, decreases in food (11.4%) and education (4.1%) were similar to those seen with inpatient treatment (p<0.001). In quartile 2, the greatest reduction occurred in construction (6.6% for Level 2 and 22.7% for Level 3, p<0.001) and education (2.9% for Level 2 and 4.0% for Level 3 p<0.001). For quartile 3, greatest reductions for Level 2 and Level 3 treatment appeared in education (3.7% and 3.5%), production means (1.4% and 4.7%), and durable goods (1.1% and 3.9%) (p<0.001). Food decreased in Level 2 (1.0%) but increased in Level 3 (2.5%). For quartile 4, significant decreases occurred in education (2.5% for Level 2 and 3.6% for Level 3, p<0.001), construction (30.9% for Level 2 and 2.8% for Level 3, p<0.001), and food (1.1% for Level 2 and 3.0% for Level 3, p<0.001). (Table 5).

### The Economic Burden of Health Expenses

A 1% increase in health expenditure is most associated with decreases in construction (0.8%) and food (0.4%) for income quartile 1; construction (4.0%) and education (0.5%) for quartile 2; construction (0.9%) and production means (0.4%) for quartile 3; and production means (3%) and construction (1.3%) for quartile 4. (Table 6).

**Table 6:** Impact of health expenses on household allocation patterns: a 1% increase in health expenditure is associated with __% change in consumption category.

		Income Quartile			
Allocation category	Total	1	2	3	4
Food	0.19****	−0.39****	−0.03****	0.04****	0.42****
Education	−0.07****	−0.24****	−0.47****	−0.10****	0.05****
Production means	−1.59****	−0.18****	−0.08****	−0.36****	−2.95****
Transportation	0.00****	−0.02****	0.00*	−0.03****	0.03****
Construction	−1.40****	−0.80****	−4.01****	−0.93****	−1.25****
Charity	−0.01****	−0.02****	0.00	0.03****	0.01****
Durable goods	−0.09****	−0.03****	−0.05****	−0.22****	−0.01****
Utilities	0.02****	−0.04****	−0.03****	0.04****	0.05****
Daily goods	0.01****	−0.04****	0.01****	0.02****	0.02****
Social activities	0.00****	0.01****	−0.02****	0.00****	0.01****
Insurance	0.00****	0.00****	0.00****	0.00**	0.00****
Gifts	0.00***	0.00****	0.00	0.00****	0.00****
Tobacco/Alcohol	0.01****	−0.03****	−0.03****	0.04****	0.01****
Loan interest	0.00****	−0.01****	0.01****	−0.08****	0.02****
Other	−0.16****	−0.03****	−0.06****	−0.39****	0.05****

*Note*: This table depicts multivariate results after controlling for household size; age, gender, marital status, occupation and education of household head; presence of household member under 18 years or over 65 years old. *Significant at *p*<0.10. **Significant at *p*<0.05. ***Significant at *p*<0.01. ****Significant at *p*<0.001.

## Discussion

Our study indicates that poorer households have disadvantages that could worsen their situation when ill. Lower income quartiles have a lower percentage of married household heads (reflecting fewer working household members) and a higher percentage of female household heads (reflecting lower income due to gender differences in capacity for physical labor). In addition, more households in lower quartiles have members older than 65 and younger than 18, yielding higher dependency ratios. Dependency ratios can inform policy-making and resource allocation [19], indicating a household’s need for affordable health treatment. With children’s education expenditure, vulnerability of dependents to illnesses, and fewer employed members, these households have higher expenses and fewer resources.

### Food Consumption

Within income quartile 1, households with inpatient and higher-level outpatient treatments faced a reduction in food consumption, which can exacerbate existing illness, threaten future health, and create more health costs. This could lead to decreased productivity in school and work, and compromise functionings such as health and financial security. When faced with inpatient treatment, higher-income quartiles did not experience the same food reductions as the poorest quartile. However, quartile 3 households with Level 2 outpatient treatment and quartile 4 households with Level 2 and 3 outpatient treatments did decrease food consumption, suggesting that even higher-income households could be forced to reduce food consumption.

### Changing Investment in Education

Households in quartiles 1 and 2 with at least one inpatient treatment showed a reduction in education expenditure, compared to households without inpatient treatment. When confronted with outpatient treatment, all quartiles decreased spending on education, with larger reductions as levels of treatment rise in quartiles 1, 2 and 4. Forced to decrease education, households lose the skills, qualifications and resources to pursue occupations and the ability to secure economic stability, an important capability. Because education in Vietnam is now paid for out of pocket and is not necessarily seen as an immediate need, households could be more likely to reduce investment in it.

### Losing Resources for Economic Stability

All quartiles with inpatient treatment reduced production means, which shows that households might be forced to decrease spending on daily farming expenses. Because outpatient costs accumulate over several visits, a household might have time to cover costs by investing in production to increase income. However, once the treatment level and costs rise, allocating resources to production means becomes more difficult. Once quartile 1 households reach Level 3 treatment, their income must go to paying health fees, and expenditures for production means decrease.

### Equity Implications of Healthcare Costs

While the mean healthcare consumption is similar between quartiles 1 and 4, health expenses constitute a much greater percentage of household allocations for quartile 1. While higher-income households can reduce purchase of expendable items to pay health costs, lower-income households lack such flexibility. Instead, they reduce more essential consumptions (food, education, and production means) that impact basic capabilities. Higher income quartiles also decrease spending in some of these areas, indicating that they are not immune to detrimental effects of health costs on basic capabilities. Their decreases were less, emphasizing the greater vulnerability of poor households.

### Comparison of Inpatient and Outpatient Treatments

While single inpatient treatments cause sudden financial shocks, cumulative outpatient treatments can have similarly detrimental effects over time. For example, for quartile 1, households show similar reductions in food consumption between inpatient and Level 3 outpatient treatment. However, findings suggest that aggregated outpatient visits have a greater effect than inpatient treatment. For example, quartile 4 households reduced food consumption for Level 3 outpatient but not for inpatient treatment. Aggregated outpatient visits (due to prolonged episodes of illness, chronic disease, or multiple sick household members) can accumulate to force households to reduce spending in other allocation categories.

### Policy Implications

This study suggests that the costs associated with health treatment force households to decrease consumption in ways that can reduce their basic capabilities. To prevent this, policy efforts should focus on reforming Vietnam’s health insurance system.

Current Vietnamese reforms focus on high hospitalization costs, and have decreased OOP by 18% [1]. However, when treatment costs are significant enough to be associated with food reduction, as shown in our study, an 18% decrease is insufficient to protect households from health shocks. Our study also demonstrates a need to broaden Vietnam’s insurance system to cover more outpatient services.

Currently only 40% of the Vietnamese population is covered by insurance [1]. Health Care Fund for the Poor (HCFP), Vietnam’s insurance scheme for low-income households, currently covers only one quarter of the eligible population [1]. Our study emphasizes the need for improved means of paying for rising health costs, and for the shift of the economic burden from the individual household to wider distribution through risk pooling across a larger population. This underscores the urgency in HCFP becoming accessible to all households in need.

Health policy must focus on achieving universal health insurance. This requires addressing the impact of healthcare expenses on all income quartiles by expanding insurance coverage and reducing OOP by covering more services.

### Limitations

Limitations to our study include recall bias. Studies have shown that it is difficult to remember household expenses after just days of consumption [20]. To attain more accurate data, we asked respondents to estimate consumption per units of time that were easiest for them to remember (for example, amount spent on food per day or week). While people may not remember exact number and cost of items, they have a general idea of budget and it may be easier to recall a general budget allocation than the exact price of an item. When recalling inpatient and outpatient services, we aimed to heighten accuracy by asking households to recall specific illness episodes for specific household members and the cost of the services and medicine needed. On a related note, recall problems may also affect the accuracy of self-reported household income from multiple itemized sources. Furthermore, there is the possibility that households might over-report or under-report income, perhaps for social or tax reasons. However, because we aggregate households into income quartiles and because commune designation of household economic status serves as a kind of external corroboration, inaccuracies in income self-reporting are unlikely to have significant effects on our results. Since the numbers of medical treatments received in the past year were self-reported as well, these figures too are vulnerable to faulty recall. This is especially true for outpatient treatments, as there were often multiple episodes of outpatient treatment per household. But as with income data, the impact of treatment episodes inaccuracies is blunted by our aggregation of outpatient treatment episodes into three levels (0–4 episodes, 5–10 episodes, >10 episodes). Most respondents are likely to be accurate within those margins.

Defining levels of outpatient treatment by the number of medical visits, however, is itself a measurement limitation. Defining intensity of treatment by number may not encompass all factors associated with financial burden, such as decreased work that would impair households’ capabilities.

A third limitation is the reporting of health treatment cost, which includes costs for a variety of items, as a single item. Two studies [21], [22] found that reporting all of these costs as a single item provides a lower estimate than reporting as an aggregate of multiple items. Neither study determined which method is more accurate. To limit the problem of possible underestimation, we based our analysis on percentages of consumption. This way healthcare and other outlays might be similarly underestimated, resulting in approximately accurate percentages of consumption.

A fourth limitation is our inability to capture the fluctuations in consumption and income over time. By analyzing the “average effects” over a 12-month period, we could overlook temporary changes in consumption immediately following a health treatment. However, temporary changes are unlikely to have major effects on households. It is the long-term change in consumption detectable in a year-long period that could worsen health and economic status.

A fifth limitation involves analysis of first-order, not second-order, effects of changes in healthcare expenditure. We describe the effect of healthcare consumption on each of the other categories (first order). We do not account for how changes in the other consumption categories affect one another following a change in healthcare consumption (second order). We believe that the first-order effect should represent the majority (if not all) of the effect of illness on consumption patterns. The second-order effects, although they do exist, are expected to be small. Other studies [10], [12] were conducted based on similar assumptions.

A sixth limitation involves caution in interpreting our findings. Our results do not show causality between health treatment and reduced consumptions. They show that health costs are associated with decreases in basic capabilities. Our study warrants further research to determine the subsequent effects of these financial decreases on health outcomes and income poverty.

Finally, because the results represented households from only one commune, they were not generalizable to the entire Vietnamese rural population. However, the study might be applicable to rural communes with demographic characteristics similar to Dai Dong.

### Conclusions

Both the immediate shock of inpatient treatment and longer-term aggregate outpatient health costs pose a substantial threat to the health and economic security of Vietnamese households, even higher-income households. Our study illuminates the implications of an unaffordable healthcare system by revealing how health expenses are associated with reductions in multiple areas of household flourishing. Healthcare reform needs to address the insufficient public spending on healthcare and the resulting reliance on OOP that fosters vulnerability and insecurity and inhibit human flourishing [23].
